# APEC Emerging Infections Network: prospects for comprehensive information sharing on emerging infections within the Asia Pacific Economic Cooperation.

**DOI:** 10.3201/eid0403.980337

**Published:** 1998

**Authors:** A M Kimball, C Horwitch, P O'Carroll, S Arjoso, C Kunanusont, Y S Lin, C Meyer, L Schubert, P Dunham

**Affiliations:** University of Washington, Seattle, USA.


### APEC Emerging Infections Network: Prospects for Comprehensive Information Sharing on Emerging Infections within the Asia Pacific Economic Cooperation

**Ann Marie Kimball,* Carrie Horwitch,* Patrick O'Carroll,† Sumarjati Arjoso,‡ Chaiyos Kunanusont,§ Ya-Shin Lin,* Clifford Meyer,* Laura Schubert,* and Phillip Dunham***

*University of Washington, Seattle, Washington, USA; †The Centers for Disease Control and Prevention, Atlanta, Georgia, USA; ‡Ministry of Health, Indonesia; and §Ministry of Public Health, Thailand

Trading blocs realize the strategic importance of and threats from emerging infections, particularly those related to travel and food. Like the European Union, the Asia Pacific Economic Cooperation (APEC) is undertaking an initiative in emerging infections.

The APEC Emerging Infections Network project builds on an existing Internet-based educational network (APEC EduNet), created to help link APEC "study centers" at designated universities. Use of collaborative tools, such as e-mail and the World Wide Web, helps bridge the broad geographic expanse and diversity of APEC economies, permitting scientists and policy makers to share information and more effectively combat emerging infectious disease through surveillance, prevention, research, and control measures.

In the project's first year, staff made site visits to Thailand, Indonesia, the Philippines, and Canada, and compiled information regarding Internet access in these selected economies. Multidrug-resistant tuberculosis (MDRTB) was selected as a disease priority by the partner economies. Accurate, prospective surveillance data on MDRTB are not generally available. Information sharing by e-mail and automated e-mail lists has been successful, and feedback suggests these strategies will become increasingly useful. The Emerging Infections Network (EINet) Web site includes project information, surveillance data, policy discussion, prevention guidelines, and distance learning resources about emerging infections.

Human networking is as important as technology-based networking in addressing emerging infections. Technology is adequate to support communications if a comprehensive telecommunications strategy is used. APEC, unlike the European Union, does not have the treaty basis to support this intercountry collaboration, so memoranda of understanding are needed to facilitate sustainable surveillance information flow and scientific cooperation. Numerous member economies are eager to be included in project activities. In the second year the project is expanding both in terms of breadth of information and geography of economies.

### Additional Information

1. Kimball AM. Pacific Rim economic ties spur emerging infections network. Washington Public Health 1997;22.
2. Lance CR, Joseph CA, Bartlett CLR. European surveillance of travel-associated Legionnaires disease. Slide presentation at the International Conference on Emerging Infectious Diseases, Atlanta.
3. WHO/IUATLD. Global project on anti-tuberculosis drug resistance surveillance, 1994-1997. Geneva: World Health Organization;1997. p. 1-227.

